# Vanquishing the computational cost of passive gamma emission tomography simulations leveraging physics-aware reduced order modeling

**DOI:** 10.1038/s41598-023-41220-3

**Published:** 2023-09-12

**Authors:** Nicola Cavallini, Riccardo Ferretti, Gunnar Bostrom, Stephen Croft, Aurora Fassi, Giovanni Mercurio, Stefan Nonneman, Andrea Favalli

**Affiliations:** 1 European Commission, Joint Research Centre, Via Enrico Fermi, 21027 Ispra, VA Italy; 2 Engineering Ingegneria Informatica, Piazzale dell’Agricoltura, 00144 Rome, RM, Italy; 3 Lancaster University, Bailrigg, Lancaster, UK; 4 Los Alamos National Laboratory, P.O. Box 1663, Los Alamos, NM 87545 USA

**Keywords:** Physics, Nuclear physics, Mathematics and computing, Scientific data, Nuclear energy

## Abstract

Passive Gamma Emission Tomography (PGET) has been developed by the International Atomic Energy Agency to directly image the spatial distribution of individual fuel pins in a spent nuclear fuel assembly and determine potential diversion. The analysis and interpretation of PGET measurements rely on the availability of comprehensive datasets. Experimental data are expensive and limited, so Monte Carlo simulations are used to augment them. However, Monte Carlo simulations have a high computational cost to simulate the 360 angular views of the tomography. Similar challenges pervade numerical science. With the aim to create a large dataset of PGET simulated scenarios, we addressed the computational cost of Monte Carlo simulations by developing a physics-aware reduced order modeling approach. This approach combines a small subset of the 360 angular views (limited views approach) with a computationally inexpensive proxy solution (real-time forward model) that brings the essence of the physics to obtain a real-time high-fidelity solution at all angular views but at a fraction of the computational cost. The method’s ability to reconstruct 360 views with accuracy from a limited set of angular views is demonstrated by testing its performance for different types of reactor fuel assemblies.

## Introduction

Passive Gamma Emission Tomography (PGET) is a measurement technique developed for spent nuclear fuel verification to help meet nuclear non-proliferation and safeguards requirements. It is part of the armory of technical measures put in place by the International Atomic Agency (IAEA), in the framework of the Treaty on the Non-Proliferation of Nuclear Weapons (NPT), to ensure that each Member State complies with the requirements of spent nuclear fuel stewardship and accountancy. The PGET system is designed to perform partial defect and bias defect verification of a spent nuclear fuel assembly while it is shielded underwater prior to being moved to long term storage or into a geological repository. The IAEA approved PGET in 2017 for inspections^1–6^.

The PGET detects the passive gamma-ray emissions from the assembly arising from the build-up of fission products generated during the use in the reactor. The most important gamma-ray signatures come from the fission products ${^{137}\textrm{Cs}}$, ${^{134}\textrm{Cs}}$, ${^{154}\textrm{Eu}}$, ${^{106}\textrm{Ru}}$, and ${^{144}\textrm{Ce}}$^7^. They are important because they confer information about the initial enrichment, burnup and cooling time of the assembly while being practical to measure. Only a few fission products are produced in sufficient quantity, and have intense yet long-lived and penetrating gamma-ray emissions to be of general interest for nondestructive assay applications.

The PGET design consists of two highly collimated linear arrays comprising up to 91 cadmium-zinc-telluride (CdZnTe) gamma detectors. The detector pitch is 4 mm. Each detector is behind a tungsten collimator 15 mm wide, 100 mm thick, and 5 mm high in front of the detector, increasing to 70 mm at the exit. The two detector heads are on a rotating disk on opposite sides with a 2 mm offset. This 2 mm stagger is devoted to obtaining 182 data collections, referred to as counts, per each view. The technical details of the PGET detector system are reported in^3, 8, 9^.

The two detector arrays rotate continuously around the spent fuel assembly. The number of events are integrated both over energy and rotation windows. Typical values for the energy windows are: < 400 keV, 400–600 keV, 600–700 keV, and 700–1200 keV, while rotations are integrated over each of the 360 degrees. The end product of a PGET acquisition sequence is a sinogram for each energy window, a matrix characterized by a number of rows equal to the number of detectors and a number of columns equal to the number of angular views, 360 in a standard acquisition. An accurate sinogram is needed as the input to tomographic reconstruction algorithms aimed at reconstructing spent fuel axial cross-sections in order to detect cases of anomalies or diversion, even to the single rod level (so-called bias defect).

It is worth mentioning that compared with medical and industrial tomography systems, PGET involves peculiar challenges due to the wide range of gamma activities that characterize the spent nuclear fuel and the high gamma attenuation of the nuclear materials. These parameters are outside the control of the experimenter and are challenging in the case of detecting anomalies in the center of the assemblies^10^.

Development and evaluation studies of algorithms for the analysis and interpretation of PGET measurements, for example, based on artificial intelligence^6, 11^, rely on the availability of extensive and comprehensive datasets of experimental and/or simulated data. Experimental data are extremely limited in availability and breadth of potential diversion scenarios, and are also expensive to generate. Realistic simulations of spent nuclear fuel measurements are therefore essential in evaluating the performance of the algorithms behind the interpretation of PGET measurements, especially in robustly identifying potential diversion situations.

Monte Carlo simulations, for example using the Monte Carlo N-Particle (MCNP) code^12^, allow the construction of a realistic and extended dataset of simulated cases and related sinograms; however, the computational cost is high. High-fidelity simulations, in fact, require the treatment of the full physics radiation transport in a detailed geometry of the PGET system. It is worth to highlight that the detection efficiency of the collimator-detector system is exceptionally low. Miller et al. and Wittman et al. pointed out that a high-fidelity MCNP simulation of the PGET measurement of a fuel assembly, the basic 360 angular views of a sinogram, take about 6–7 days in a cluster composed of 128 nodes and 8192 cores^4, 13, 14^.

Our objective is to create an extensive library of PGET scenarios. According to^14^, thousands of simulated scenarios are needed. To produce simulated cases, we addressed the issue of the high computational cost by developing a physics-aware reduced-order modeling approach. In this approach we combine (1) a limited number of the 360 angular views (limited views tomography) and (2) a real time analytically-built approximated sinogram (based on the Lambert-Beer law) at all the 360 views, that brings the essence of the physics. The computational cost is cut by only simulating a sparse subset of angular views. The computational benefit is proportional to the number of skipped views (e.g., simulating 60 views instead of 360 reduces the cost to about one-sixth). The Physics-Aware Reduced Order Model approach then enables the reconstruction of all the angular views. This is achieved using Proper Orthogonal Decomposition (POD)^15–18^, a subset of the techniques available in Reduced Order Modeling numerical methods, which identifies the dominant patterns in the data by applying Singular Value Decomposition (SVD) to the matrix of the limited views data. We named our method Physics-Aware Proper Orthogonal Decomposition (PA-POD).

The method’s performance is tested and measured against the data released by IAEA under the PGET Tomographic and Analysis Challenge (2019)^19, 20^. We used the IAEA field dataset as surrogate of realistic simulations. The dataset is used as the ground truth to which apply our PA-POD method. We sample limited set of angular views, and we reconstruct the sinogram at each angular view via PA-POD. The goodness of the method is obtained by comparison between ground truth and the reconstruction. Among the collection of IAEA data, three cases are of particular interest because they refer to field scenarios. They are named competition three, four, and five, and related to a VVER (water-water energetic reactor), a PWR (Pressurized Water Reactor), and a BWR (Boiling Water Reactor) fuel assembly, respectively. In the Section “Results” we report the results of the PA-POD method applied to the IAEA PWR assembly case, as PWR nuclear plants are the large majority of nuclear plants in the world. The result of the VVER and BWR IAEA fuel assemblies are provided in the Supplement Material. All the results presented refer to the 600–700 keV window: it contains the ${^{137}\textrm{Cs}}$ gamma emission line of 661.7 keV, a primary gamma emission of spent nuclear fuel with a relatively long half-life of 30 years^7^.

The paper is organized as follows. The first Section, “Our approach” explains our PA-POD method PA-POD and its foundations; the second Section, “Results: the PWR case” reports the results of the method applied to the PWR case. The third Section, “Discussion” compares the method with other approaches and discusses the enhancement to the PWR challenge data. The fourth Section, “Methods” reports details of the implementation of our PA-POD approach. Supplementary material reports the results for the BWR and VVER cases.

## Our approach

We consider the sinogram matrix ${\textbf{S}}$, our ground truth, as a discrete representation of an unknown continuous solution ${\textbf{s}}(y,\theta )$, where *y* represents the spatial coordinate of the detector and $\theta$ is the angular coordinate. POD approximates the solution as a linear combination of dominant patterns, or modes, ${{\textbf{u}}_i(y)}$ estimated using only a reduced set of angular views. The approximated solution is:

1 $$\begin{aligned} {\tilde{\textbf{s}}}(y,\theta )=\sum _{i=0}^{k-1} {\textbf{u}}_i(y)\, {\textbf{c}}_i (\theta ), \end{aligned}$$

with *k* the number of selected modes. All the possible linear combination of the modes $\{{\textbf{u}}_i(y)\}$, with $i=0,\ldots ,k-1$, construct the so-called POD solution space (or POD space), where *k* is its dimensionality. In the following steps, we fix the notation and summarize our PA-POD approach.

1. *Database Creation* Take the set of $N_s$ simulated/measured limited angular views at the angular coordinates $\theta _n$ and construct the set of pairs $\{{\textbf{s}}(y,\theta _n),\theta _n\}$, with $n = 0,\ldots ,N_s-1$. They are stored in a database matrix ${\hat{\textbf{S}}}_{N\times N_s}$, where *N* is the number of detectors.
2. *POD Solution Space Construction* Apply Singular Value Decomposition to the database matrix: ${\hat{\textbf{S}}} = {\textbf{U}}\,{\varvec{\Sigma }}\, {\textbf{V}}^*$. Meaning decompose the database matrix into two unitary matrices ${\textbf{U}}$ and ${\textbf{V}}$ and a diagonal matrix ${\varvec{\Sigma }}$. Here $^*$ denotes the complex conjugate transpose. The diagonal elements $\delta _j$ of ${\varvec{\Sigma }}$, or singular values, are non-negative and ordered from the largest to the smallest. They are usually normalized in the form $\sigma _j = \frac{\delta _j}{\sum _{l=0}^{N- 1}\delta _l}$. We define information variance relative to the *j*-th singular value as the sum of all the first $\sigma _j$. It is a measure of how much of the total data variability is captured by the first *j* modes^18, 21^. The *i*-th column of ${\textbf{U}}$ stores the mode ${\textbf{u}}_i(y)$. To construct a suitable POD solution space we balance between the number of modes and the information variance. This reduces the dimensionality of the problem and avoids selecting higher modes^21^ which are generally associated with noise rather than information.
3. *Coefficients Estimation* We use $\mathbf {U^*}_{k\times N}$, the conjugate transpose of the first *k* columns of the modes matrix ${\textbf{U}}$, to project into the POD space ${\textbf{R}}_{N\times 360}$, a computationally inexpensive approximation of the full sinogram ${\textbf{S}}$. We refer to this approximation as the Real-Time Approximate Forward Model, shortened as Real-Time Model. In the PGET case it consists in a Lambert exponential attenuation model applied to a voxelization of the fuel assembly geometry. It simplifies the gamma photon transport and enables a real time approximation at any possible PGET angular view. This model was initially proposed by^9^, details on our own implementation are described in Section “Methods”. As a result we obtain the estimation of the set of coefficients at the 360 views, $\{{\textbf{c}}_i(\theta _m)\}$ in Eq. (1), with $i = 0,\ldots ,k-1$, and $m = 0,\ldots ,359$. In matrix form: ${\tilde{\textbf{C}}}_{k\times 360} = \mathbf {U^*}_{k,N}\, {\textbf{R}}_{N\times 360}$. The coefficients $\tilde{\mathbf {{C}}}$ are row wise scaled to match ${\hat{\textbf{S}}}$ values.
4. *Solution Evaluation* The final approximated sinogram is represented by Eq. (1), that can be written in matrix form: ${\tilde{\textbf{S}}}={\textbf{U}}\, {\tilde{\textbf{C}}}$.

We define and report the error on the reconstructed image rather than on the sinogram because in the PGET analysis the sinogram quality is judged by the quality of its reconstruction. As a metric we choose to quantify the error matrix ${\textbf{e}}$ of the filtered back projection (FBP, mathematically represented with the operator ${\mathscr {F}}(\cdot )$) applied to the ground truth, to the Real-Time Approximated Forward Model, and to our PA-POD estimation. Specifically, we used the ramp-filtered version of FBP^22, 23^. In explicit terms, we have the following pixel wise error map definitions:

$$\begin{aligned} {\textbf{e}}_{\mathrm {Real-Time\ Model}} = \left| \frac{{\mathscr {F}}({\textbf{R}})-{\mathscr {F}}({\textbf{S}})}{{\mathscr {F}}({\textbf{S}})}\right| ;\quad {\textbf{e}}_{\mathrm {PA-POD}} = \left| \frac{{\mathscr {F}}({\tilde{\textbf{S}}})-{\mathscr {F}}({\textbf{S}})}{{\mathscr {F}}({\textbf{S}})}\right| . \end{aligned}$$

We apply a mask (matrix) to select the ${\mathscr {F}}({\textbf{S}})$ values that are greater than 15% of the maximum. The mask covers the assembly cross section and its premises, this area is addressed as $\Omega _{\textrm{tot}}$.

Furthermore, we use the cumulative error distribution as an integral error measure of maps ${\textbf{e}}_{\mathrm {Real-Time\ Model}}$ and ${\textbf{e}}_{\mathrm {PA-POD}}$. Given an error threshold $\epsilon _{\textrm{th}}$, we count the number of pixels with a smaller or equal error, we label it with $\Omega |_{\epsilon _{\textrm{th}}}$. We define the pixel fraction as the ratio between this area and $\Omega _{\textrm{tot}}$:

2 $$\begin{aligned} \mathrm {pixel\ fraction}=\frac{\Omega |_{\epsilon _{\textrm{th}}}}{\Omega _{\textrm{tot}}}, \end{aligned}$$

the greater its value, the better the performance. The metric we use in this work does not require any arbitrary filtering as others of the commonly used metrics in literature (see, for example, the Structural Similarity Index^24^).

## Results: PWR case

**Figure 1:**
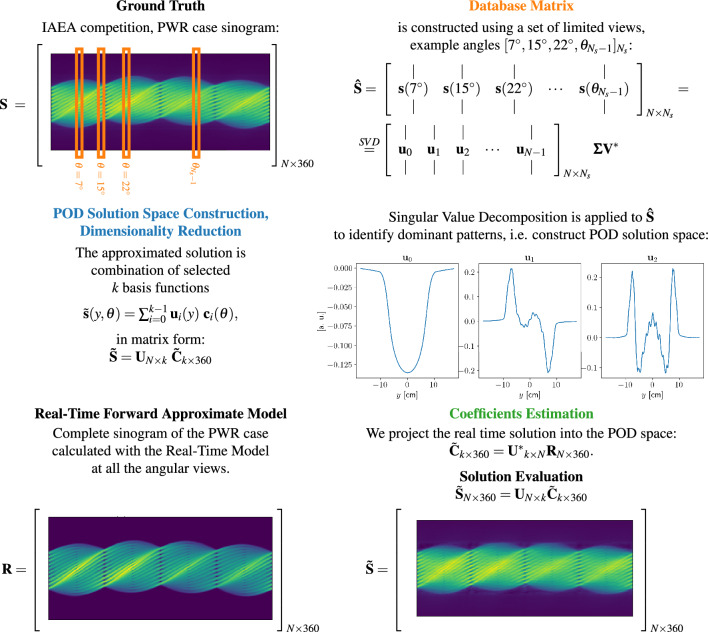
The figure reports the major steps in our PA-POD method. On top left we represent the actual sinogram for the PWR assembly from the IAEA competition (our ground truth), as high fidelity data, named ${\textbf{S}}$ in our formulation. With the orange color we highlight the subset of limited views that construct the database matrix. We present the POD solution space and plot the first three modes ${\textbf{u}}_0, {\textbf{u}}_1, {\textbf{u}}_2$ for the case of $N_s = k = 60$ randomly chosen views. On bottom left, the sinogram ${\textbf{R}}$, on the same pin configuration, as obtained by the Real-Time Approximate Forward Model. At the bottom right the PA-POD formulation and resulting sinogram $\tilde{{\textbf{S}}}$.

The following results report the application of our PA-POD approach, starting from a sparse sampling of the 360 angular views of the PWR assembly sinogram included in the IAEA competition dataset. Specifically, it consists of a 10$\times$10 pins assembly where a 3$\times$3 block of pins has been removed; thus it is also a case of a diversion scenario. The Real-Time Model relies on the investigation domain discretisation, see Section “Methods” for the details, the reported results are computed with the following mesh sizes: $\Delta z =10$ mm, $\Delta x =\Delta y =0.5$ mm, being *x*, *y*, *z* the three coordinate axes of the investigation domain. Both the ground truth sinogram and the Real-Time one are normalized between zero and one.

The flowchart in Fig. 1 summarises our method and presents an overview of the results of the PWR case. In particular, we report the ground truth sinogram ${\textbf{S}}$, the real-time approximated sinogram ${\textbf{R}}$, the first three modes ${\textbf{u}}_0, {\textbf{u}}_1, {\textbf{u}}_2$ and the PA-POD approximate sinogram $\tilde{{\textbf{S}}}$. In the real-time approximated forward model, we assume the intensity of pins of the PWR assembly is flat where the pins are located, and zero in the 3 by 3 block in the lower left center where the pins are removed. The PA-POD sinogram $\tilde{{\textbf{S}}}$ is constructed randomly sampling 60 views for the database matrix, and the first 60 modes are selected to construct the POD space. The orange views highlighted in the top left corner have the objective of proving a visualisation of the database matrix, the rotation angles are just examples.

**Figure 2:**
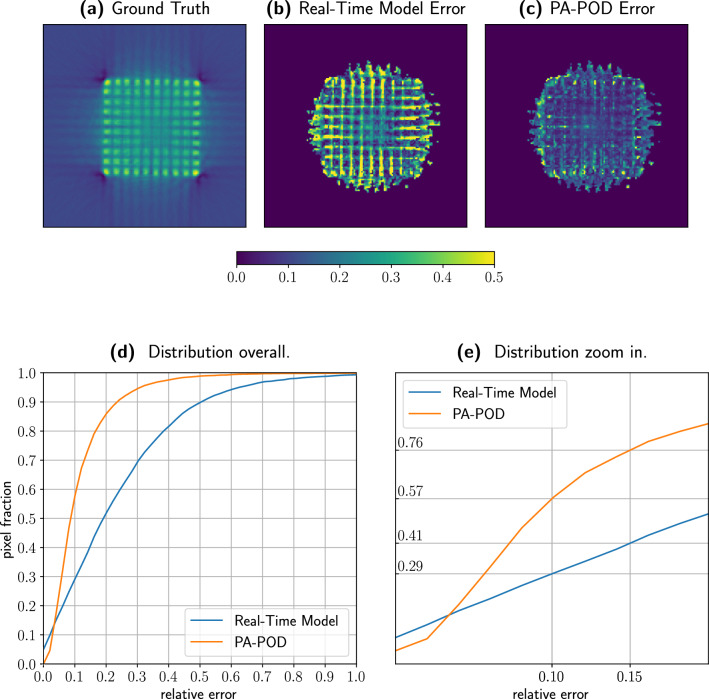
Here we represent the ground truth. (**a**) together with the error that characterizes the real time model (**b**) and our PA-POD approximation (**c**). In the PA-POD case we randomly sample sixty views of the spent fuel, we repeat the sample one hundred times, and for each pixel, we collect the median of the sampled data. (**d**) The improvement from the Real Time Model to PA-POD is quantified by the area between the blue and orange lines. (**d**) More precisely, considering a 10% error, PA-POD describes 57% of the total area, while the Real Time Model describes 29% of the area.

Figure 2 represents the error and quantifies it. In Fig. 2a the ground truth, while the error is spatially visualized in the maps Fig. 2b,c. To avoid any procedural biasing effect, we repeat the views sampling 100 times, and at each pixel we plot the median value for the error. Figure 2c shows that PA-POD can correct most of the high error pixels in between pins and around the assembly. The PA-POD relative error map clearly indicates that the approach reproduces the actual data within a relative error of the order of 10%.

Figure 2d,e complete the picture plotting the error cumulative distribution, that we called pixel fraction to emphasize its geometrical meaning, see Eq.  2. Figure 2d starts with a minimal number of pixels having the same value as the ground truth due to the normalization of the two sinograms. As soon as we consider larger errors, the area that PA-POD can describe with a specific accuracy increases with a far steeper gradient than the Real-Time Model. Figure 2b, shows the results with a finer-graded detail. We focus on the 10% relative error and observe that PA-POD covers 58% of the total area, while the Real-Time Model describes 29% with the same accuracy. These results show that it is possible to approximately double the accuracy of the real-time model using 16% of the total number of views.

**Figure 3:**
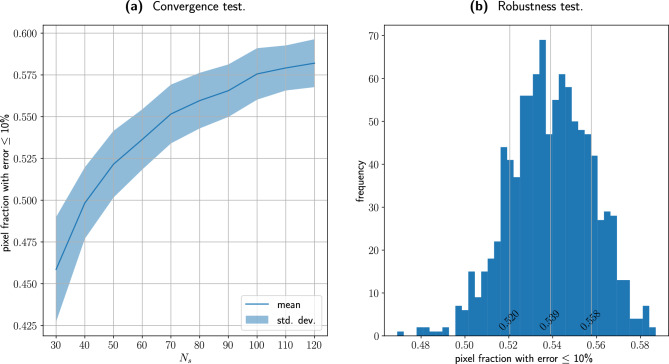
(**a**) With $k = N_s$, we vary $N_s$ from 30 to 120, with a spacing of 10 units. For each value of $N_s$ we select 100 random uniform sets of views, evaluate the pixel fraction at 10% and report the mean and the standard deviation for each distribution. The method delivers its best performance between 60 and 80 samples. In (**b**) a closer view of the pixel fraction distribution with respect the sample choice. With $N_s = k = 60$ we pick 1000 random uniform sets of views, evaluate the 10% pixel fraction for each set and plot the distribution. We observe that the mean is 0.539 and the standard deviation is 0.019, both the measures are expressed in terms of pixel fraction.

The convergence of the PA-POD method is studied in further detail in Fig. 3a. With $k = N_s$, we vary $N_s$ from 30 to 120, with a spacing of 10 units. For each value of $N_s$ we select 100 random uniform sets of views, evaluate the pixel fraction at 10% and report the mean and the standard deviation for each distribution. PA-POD expresses its best performance where the sample is very sparse, in the range 30–60 samples. From 80 samples onwards the convergence reaches a plateau, this behavior is due to the non-interpolatory nature of the coefficients, see Section “Discussion” for details. Furthermore we notice that the standard deviation is smaller than 0.02 expressed in pixel fraction at 10%, this implies that the performance is consistent with respect the views choice. In Fig. 3b we present a closer view of the pixel fraction distribution with respect the views choice. We consider the pixel fraction at 10% error and we evaluate it for 1000 uniformly random sets of sixty views, with $N_s = k=60$. The mean is 0.539 and the standard deviation is 0.019 expressed in pixel fraction. This confirms the method is robust with respect the views selection. In both Fig. 3a,b we see that the mean values for the pixel fraction at the 10% error are slightly smaller compared to the values in Fig. 2, 0.539, compared to 0.57. Evaluating the error pixel by pixel, repeating and collecting the median, as in Fig. 2, has a minimal regularization effect on the error metric, without any consequence for our overall conclusions.

**Figure 4:**
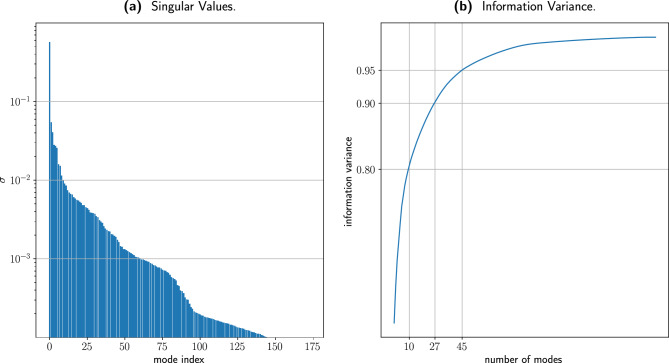
(**a**) Logarithmic plot of the singular values spectrum for the IAEA PWR fuel assembly sinogram, and (**b**) the related information variance. We highlight that it takes respectively 8, 27, and 45 modes to capture 80%, 90% and 95% of the total information variance.

Figure 4 plots the singular values for the PWR ground truth sinogram Fig. 4a and the corresponding information variance Fig. 4b. Figure 4b highlights that a reduced set of modes captures the majority of the information variance. In particular 8, 27, and 45 modes capture 80%, 90% and 95% of the total information variance respectively. This result confirms the underlying assumption of our work: a sinogram, experimentally measured or numerically computed, is described by a limited set of dominant patterns, that carefully combined together can provide a complete solution with a sparse set of angular views.

In addition, we applied our PA-POD approach to the BWR and VVER IAEA data (see Section “Methods”) and the results are reported in the supplementary material. Error maps and error distributions confirm that results are in the same range as the PWR.

## Discussion

Our approach was in part inspired by a particular flavor of Proper Orthogonal Decomposition that uses interpolation to estimate the coefficients, namely POD with Interpolation (PODI). In this domain of interest, the research effort is devoted to finding the most accurate interpolator for each applicable case^15–18^. PODI is particularly appealing because it is agnostic of the underlying physics of the phenomenon, it has no notion of the mathematical method describing the prototype, in Reduced Order Modeling literature these methods are called non-intrusive^18^. In this section we will briefly summarize the steps that define the method, we will show its limitations in the PGET case, and how we addressed them while developing PA-POD.

In PODI the interpolatory coefficients are estimated by projecting the database matrix onto the POD space: ${\textbf{C}} = \mathbf {U^*}\, {\hat{\textbf{S}}}$. The columns of ${\textbf{C}}_{k\times N_s}$ construct a set of pairs $\{{\textbf{c}}_j,\theta _j\}$, with $j = 0, \ldots , N_s-1$. Consider ${{\tilde{\theta }}}$ a rotation angle not included in our sample, we interpolate the coefficients $c_{ij}$ to get $\{\tilde{{\textbf{c}}},{\tilde{\theta }}\}$ and reconstruct:

$$\begin{aligned} {\tilde{\textbf{s}}}(y,{\tilde{\theta }})= \sum _{i=0}^{k-1} {\textbf{u}}_i(y) \cdot \tilde{{\textbf{c}}}. \end{aligned}$$

${\tilde{\textbf{C}}}_{k\times 360} = {\mathscr {I}}({\textbf{C}}_{k \times N_s})$ is the coefficients matrix for the full 360 degrees, it is the result of the interpolation operator ${\mathscr {I}}$ applied to ${\hat{\textbf{S}}}$ projected into the POD space.

**Figure 5:**
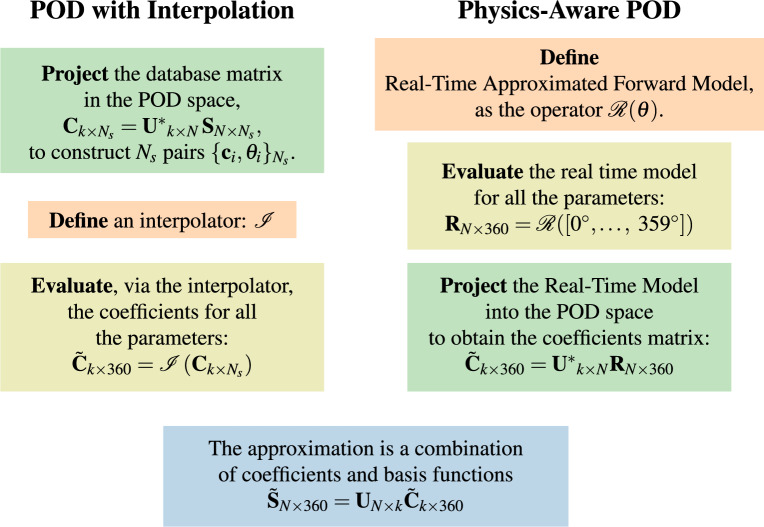
This picture shows a side by side comparison between the PODI and PA-POD. In PODI we interpolate accurate but scarce data, while with PA-POD we evaluate dense real time approximated data.

We compare the PODI and PA-POD approaches side by side in Fig. 5. In both cases we look for the same solution structure:

$$\begin{aligned} {\tilde{\textbf{S}}}_{N\times 360} ={\textbf{U}}_{N\times k}\tilde{{\textbf{C}}}_{k\times 360} \end{aligned}$$

In both cases, the coefficients are evaluated by projecting available data into the POD space:

$$\begin{aligned} {\textbf{C}}_{k\times N_s}=\mathbf {U^*}_{k\times N}\,{\hat{\textbf{S}}}_{N\times N_s} \quad \mathrm {vs.} \quad \tilde{{\textbf{C}}}_{k\times 360} =\mathbf {U^{*}{}}_{k\times N}{\textbf{R}}_{N\times 360}, \end{aligned}$$

while PODI interpolates accurate but scarce data ${\hat{\textbf{S}}}_{N\times N_s}$, conversely, PA-POD relies on approximate but dense data provided by the Real-Time Model ${\textbf{R}}_{N\times 360}$.

**Figure 6:**
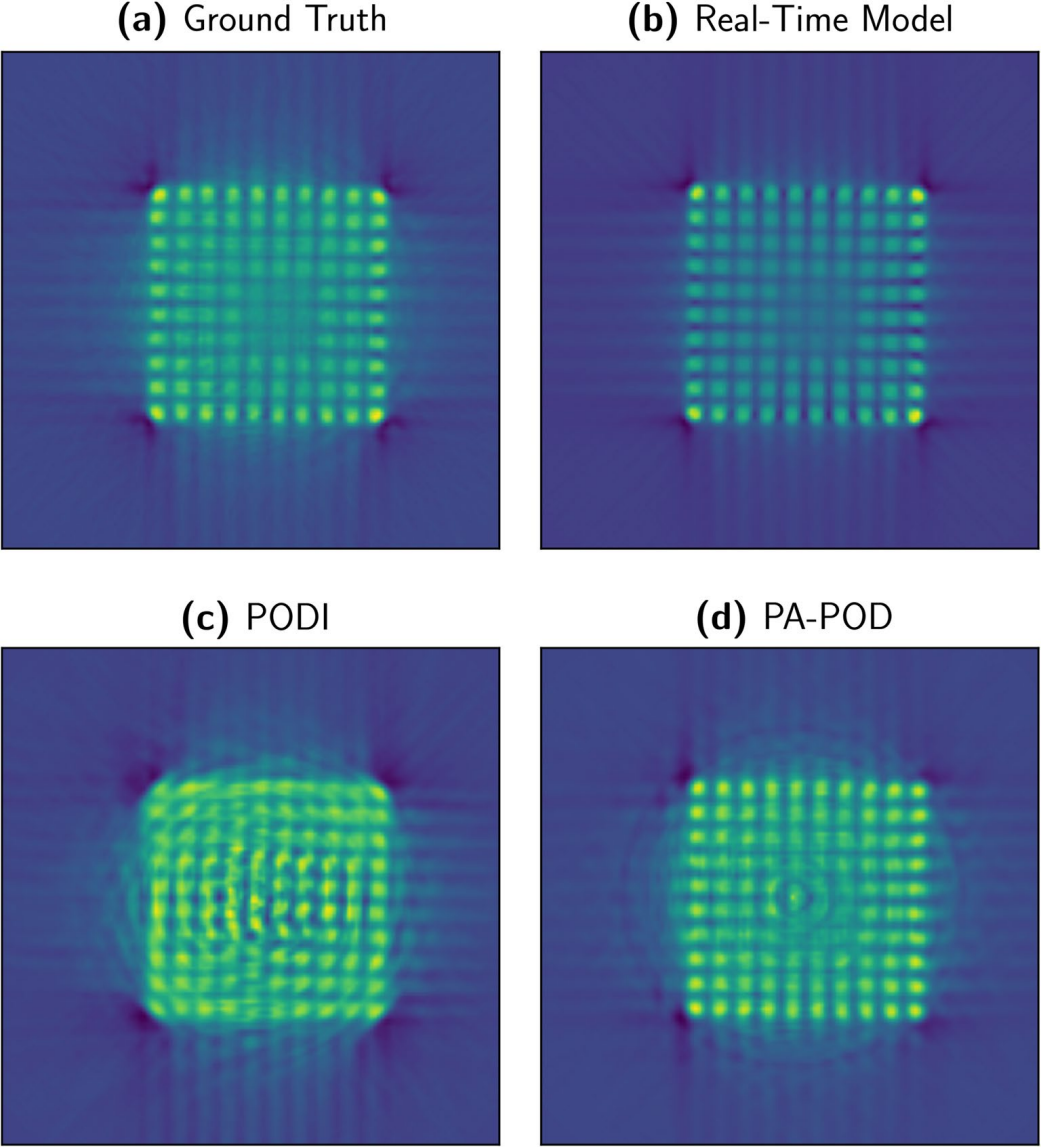
Filtered backprojection for the IAEA data (**a**), and the approximations we are studying, $N_s = k = 60$. Real-Time Model (**b**) and PA-POD **(d)** preserve the geometrical structure of the spent fuel, while PODI cannot provide a representative reconstruction (**c**).

In Fig. 6 we compare the FBP for several approximation techniques with the ground truth, (**a**). The Real-Time Model Fig. 6b is an effective approximation in describing the assembly geometry. Figure 6c,d are constructed with $N_s = k = 60$. PODI fails to capture the structure of the fuel assembly Fig. 6c, while PA-POD preserves its geometrical structure Fig. 6d and recovers part of the secondary effects in the ground truth. Ring artifact is notable in the PA-POD reconstruction, Fig. 6d, this effect can be removed using the algorithm provided in reference^25^.

**Figure 7:**
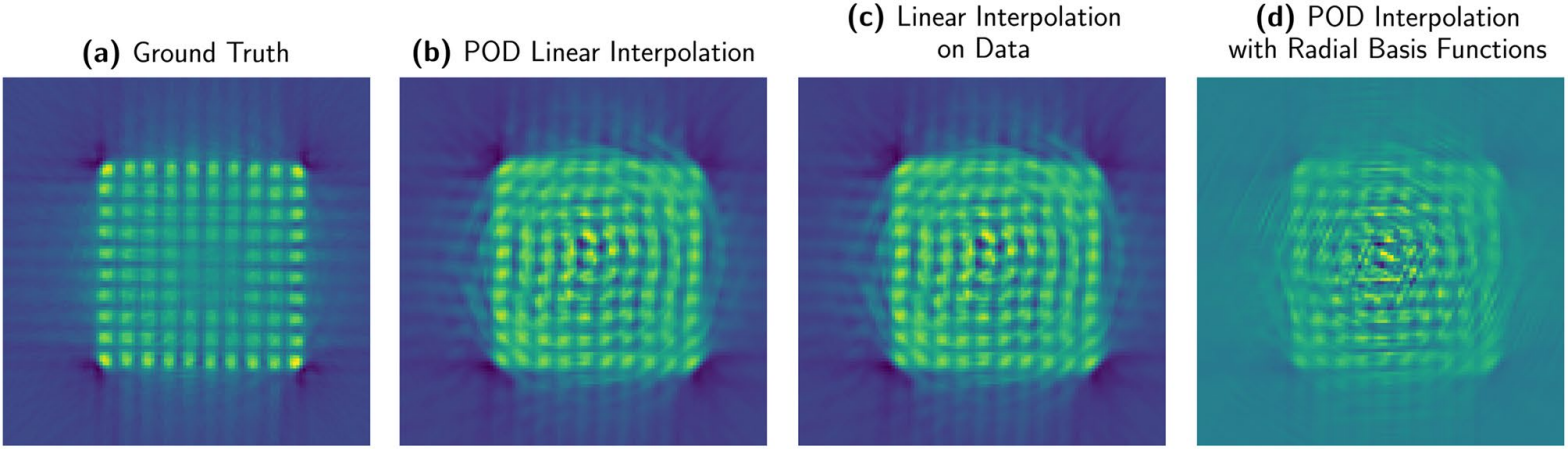
This picture presents the filtered backprojection applied to three different interpolations, with $N_s =60$ and $k =30$. (**a**) Ground truth; (**b**) POD with linear interpolation of the coefficients; (**c**) Linear interpolation on the data; (**d**) POD with radial basis functions interpolation. The geometrical structure of the fuel assembly is severely compromised in all of three approximations. None of them preserves the assembly pin structure.

We tested three different interpolators. The results are reported in Fig. 7, the ground truth in Fig. 7a,b linear interpolation on the coefficients in the POD space, Fig. 7c linear interpolation on the data, Fig. 7d radial basis functions interpolation on the coefficients, but none could preserve the assembly geometry for $N_s = k = 60$. In this phase of the exploration we relied on the EZyRB package developed at Scuola Internazionale Superiore Studi Avanzati^26^.

**Figure 8:**
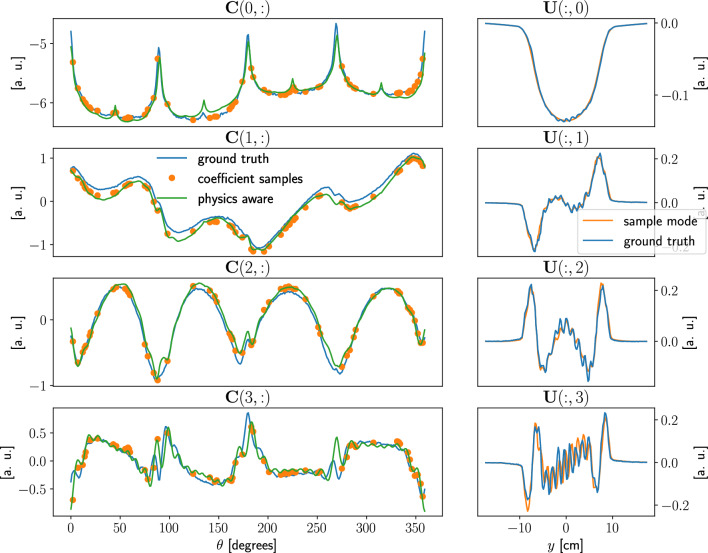
Plots of the first four coefficients ${\textbf{C}}(i,:)$ and modes ${\textbf{U}}(:,i)$ for the PWR sinogram, with $k = N_s = 60$. A few discrepancies are visible in the sampled coefficients, while the physics-aware ones have a better definition especially in areas characterized by high oscillations. The sampled modes, obtained applying SVD to ${\hat{\textbf{S}}}$, are in good agreement with ones obtained using the whole dataset ${\textbf{S}}$.

**Figure 9:**
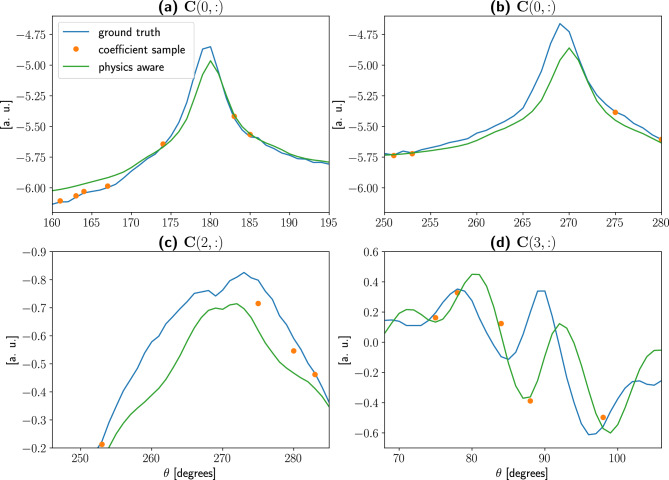
In these plots a close up view of the ground truth coefficients, the sampled ones, and the physics-aware ones are reported. Given $N_s=k=60$ with randomly sampled views, we observed that the sampled coefficients do not capture enough data to account for the local structure. The information dropped during the sampling is in fact irrecoverable, unless we provide a physics-aware way to reconstruct the coefficients. It is this notion of “physics awareness” coefficients that inspired the main idea in this article.

The most important reason why interpolating methods show an inadequate performance is the sampling scarcity. In particular, Figs. 8 and 9, show that the interpolation points fall too far apart to be effectively interpolated.

Figure 8 gives an overview of modes and coefficients, with $k = N_s = 60$. It shows the first four coefficients evaluated in three different ways. First, we project the ground truth into the POD space:

$$\begin{aligned} {\textbf{C}}_{\mathrm {ground\ truth}} = {\textbf{U}}^*\, {\textbf{S}}, \end{aligned}$$

this is the best possible approximation of the expected coefficients. Second, we project the limited views sinogram ${\hat{\textbf{S}}}$ into the POD space, as prescribed by the PODI approach, these coefficients samples are to be connected by a suitable interpolator. Third, we evaluate the coefficients with the PA-POD method, projecting into the POD space an inexpensive approximation of the full sinogram, namely the result of the Real-Time Model.

We notice that while the coefficients samples preserve an overall fitting with the ground truth, important details are missed, causing the failure of the geometrical reconstruction. On the other hand, PA-POD coefficients are not interpolatory as in the PODI case but provide an accurate description of the assembly geometry. We notice that the modes ${\textbf{U}}(:,i)$ for the database matrix ${\hat{\textbf{S}}}$ are in good agreement with the ones obtained from the full sinogram ${\textbf{S}}$, Fig. 8.

Figure 9 shows several details of the coefficient matrices. The coefficients samples are simply too far apart to capture the actual shape of the coefficient, regardless of the of the interpolator selection, there is no way to recover information that is not in the data unless physics awareness of the prototype is restored, as we did with PA-POD.

It is evident, as depicted in Fig. 9, that PA-POD coefficients are not interpolatory. As a consequence, the resulting sinogram is not exactly the original one at the sampled locations.

What is described in this section with reference to PWR remains valid for BWR and VVER cases, as reported in the supplementary material. As a consequence the method is robust with respect to the assembly type.

## Future directions

The results of PA-POD performance for the PWR case as well as BWR and VVER cases (see supplementary material) demonstrate the ability of the PA-POD method to reconstruct with high fidelity a full sinogram from a limited set of views.

This allows the construction of an extended library of various PGET measurement scenarios. The performance of our PA-POD approach enables the construction of a digital twin, where actual measurements and simulated data are combined with a deep learning algorithm in a continuous update as measurements and simulations are produced in real-time. The benefit of doing so is to provide an integrated system, hardware, and digital twin for real-time high-accuracy verification of irradiated nuclear fuel assemblies using PGET. Our proposed approach can be readily adapted and used for other applications in nuclear safeguards and beyond, such as the creation of a dataset for the Tomographic Gamma Scanner technique^27^, which is a non-destructive assay technique, as well as in other radiography/tomography approaches for nuclear material and global security applications^28^.

## Methods

### Reference data used in the paper (IAEA competition)

Our method’s development and the associated verification have benefited from high-fidelity data from real case scenarios. The dataset provided by the International Atomic Energy Agency on the occasion of IAEA Tomographic and Analysis Challenge fit the purpose. The data are publicly available^19, 20, 29^. Among several mockup data, few measurements are provided that were used in the paper. In the paper, the original names of the IAEA data, “competition 3”, “competition 4”, and “competition 5” are renamed into VVER, PWR, BWR respectively. VVER and BWR are reported in the supplement material. In our presented results, we focused on the 600–700 keV energy-deposition window, which contains the full energy peak from the ${^{137}\textrm{Cs}}$ gamma emission at 661.7 keV, a major gamma emission of the spent nuclear fuel^7^.

### Real-time approximate forward model

**Figure 10:**
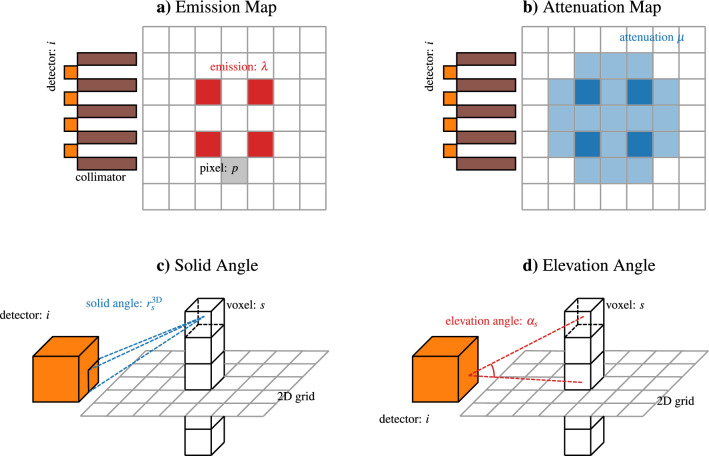
A schematic view of the major players in the real time model implementation.

Our Real-Time Model is built on the model of Backholm et al.^9^. A schematic representation of the model set is depicted in Fig. 10. The investigation domain $\Omega \subset {{\mathbb {R}}^2}$ is the axial cross-section of a nuclear fuel assembly and we discretize it as a two-dimensional mesh. At each pixel *p* of the discretized domain, we define $\lambda \ \in {\mathbb {R}}^{N_{\textrm{pix}}}$ the emission values and the attenuation values $\mu \ \in {\mathbb {R}}^{N_{\textrm{pix}}}$, $N_{\textrm{pix}}$ is clearly the number of pixels. We consider the values of $\lambda$ and $\mu$ constant over each pixel. At each detector *i* we model the flux using a Lambert-Beer (exponential) attenuation model^9^:

$$\begin{aligned} F(\lambda ,\mu )_i = H(\mu )_{i,p}\, \lambda _{p} = \left[ r_{i,p}\,\textrm{exp}\left( -c_{i,p}\, d^T_{i,p}\, \mu \right) \right] \, \lambda _{p}. \end{aligned}$$

The 360 views are obtained by rotating the pin positions, and so the assembly, with respect to the detectors. The emission and attenuation maps are consequently updated. The two-dimensional grid is further discretized, dividing the half-space above and below each pixel, in voxels.

Given this setting, the following quantities are defined.

- $r_{i,p}$ is the response of the detector *i* with respect to the pixel *p*. Given a three-dimensional point, the barycenter of a voxel *s*, $r^{3D}_s$ is the solid angle between the voxel barycenter and the detector face, divided by $4\pi$. Follows $r_{i,p}$ is the average of the responses for all the voxels, insisting on the same pixel: $r_{i,p} = \frac{1}{N_{p,\textrm{vox}}}\sum _{s=1}^{N_{p,\textrm{vox}}} r^{3D}_s$.
- $d^T_{i,p}\, \mu$ is the integral of the discrete attenuation values on the segment connecting the pixel *p* and the detector *i*. In our implementation we explicitly find the intersection between the connecting segment and the grid, being the attenuation piecewise constant, a zero-order quadrature is sufficient to exactly evaluate the integral.
- $c_{i,p}$ is an attenuation correction factor, evaluated as: $c_{i,p} = \frac{1}{r_{i,p}}\sum _{s=1}^{N_{p,\textrm{vox}}} \frac{r^{3D}_s}{\textrm{cos}(\alpha _s)}$. $\alpha _s$ is the angle between the voxel barycenter, the detector and the two dimensional plane. It accounts for the response matrix $r_{i,p}$ and the correction term $c_{i,p}$ are purely geometrical measures and are not affected by the rotation of the fuel assembly. $d^T_{i,p}\, \mu$ instead depends on the attenuation map, which depends on attenuation distribution, that changes with the assembly rotation and has to be assembled for each rotation angle.

We tested three different mesh sizes, while $\Delta z = 10\ \textrm{mm}$ was the same in all the three cases, the other dimensions are $\Delta x = \Delta y = (2.5 \ \textrm{mm}, 1.0 \ \textrm{mm}, 0.5 \ \textrm{mm})$. With a Mac Book Air computer with ARM M2 processor with four physical cores, the average computational times are $\approx 4\ \textrm{minutes}$, $\approx 10\ \textrm{minutes}$ and $\approx 31\ \textrm{minutes}$ respectively. The mesh size does not affect the accuracy of our results, the coarsest mesh size can be chosen to minimize the computational cost.

Values for emission $\lambda$ attenuation $\mu$ are defined in^9^. Emission is expressed in arbitrary units, and is 0 for water and 100 for the spent fuel. Attenuation is $0.1356\ \textrm{mm}^{-1}$ for the spent fuel, and $0.0085\ \textrm{mm}^{-1}$ for water. Virta et al. in^10^ provide an extensive exploration of such parameters in experimental settings. An approximation of the Real-Time approximate forward model is that it does not model the downscattering of the gamma rays.

The computational cost for a full sinogram according to this ray-tracing scheme is a few minutes on a desktop machine, and the assembly of all the matrices is a type of “embarrassingly parallel” task^30^, it can be parallelized both on a “per detector” basis or on a “per degree” basis. 

### Supplementary Information

Supplementary Figures.

## Data Availability

The datasets analyzed during the current study were publically distributed by the International Atomic Energy Agency on the occasion of the Tomographic and Analysis Challenge. The address for request is provided in^19^.
