# Supplementary material for: Vanquishing the computational cost of passive gamma emission tomography simulations leveraging physics-aware reduced order modeling
Source: Sci Rep. 2023 Sep 12;13:15034. doi: 10.1038/s41598-023-41220-3 (PMC10497611; doi:10.1038/s41598-023-41220-3)
Supplement: Supplementary file 1 — Supplementary Figures. [file 41598_2023_41220_MOESM1_ESM.pdf]

Supplementary Material to the paper: Vanquishing the computational cost of passive gamma emission tomography simulations: a physics-aware reduced order modeling approach

Nicola Cavallini[1], Riccardo Ferretti[1], Gunnar Bostrom[1], Stephen Croft[2], Aurora Fassi[1], Giovanni Mercurio[1], Stefan Nonneman[1], and Andrea Favalli[1,3,*]

[1]European Commission, Joint Research Centre, Via Enrico Fermi, Ispra, I -21027 (Va), Italy
[2]Lancaster University, Bailrigg, Lancaster, UK
[3]Los Alamos National Laboratory, P.O. Box 1663, Los Alamos, NM 87545, USA
[*]andrea.favalli@ec.europa.eu

March 6, 2023

VVER assembly, error maps and cumulative distribution, for, $N_s = 60$, $k = 60$.

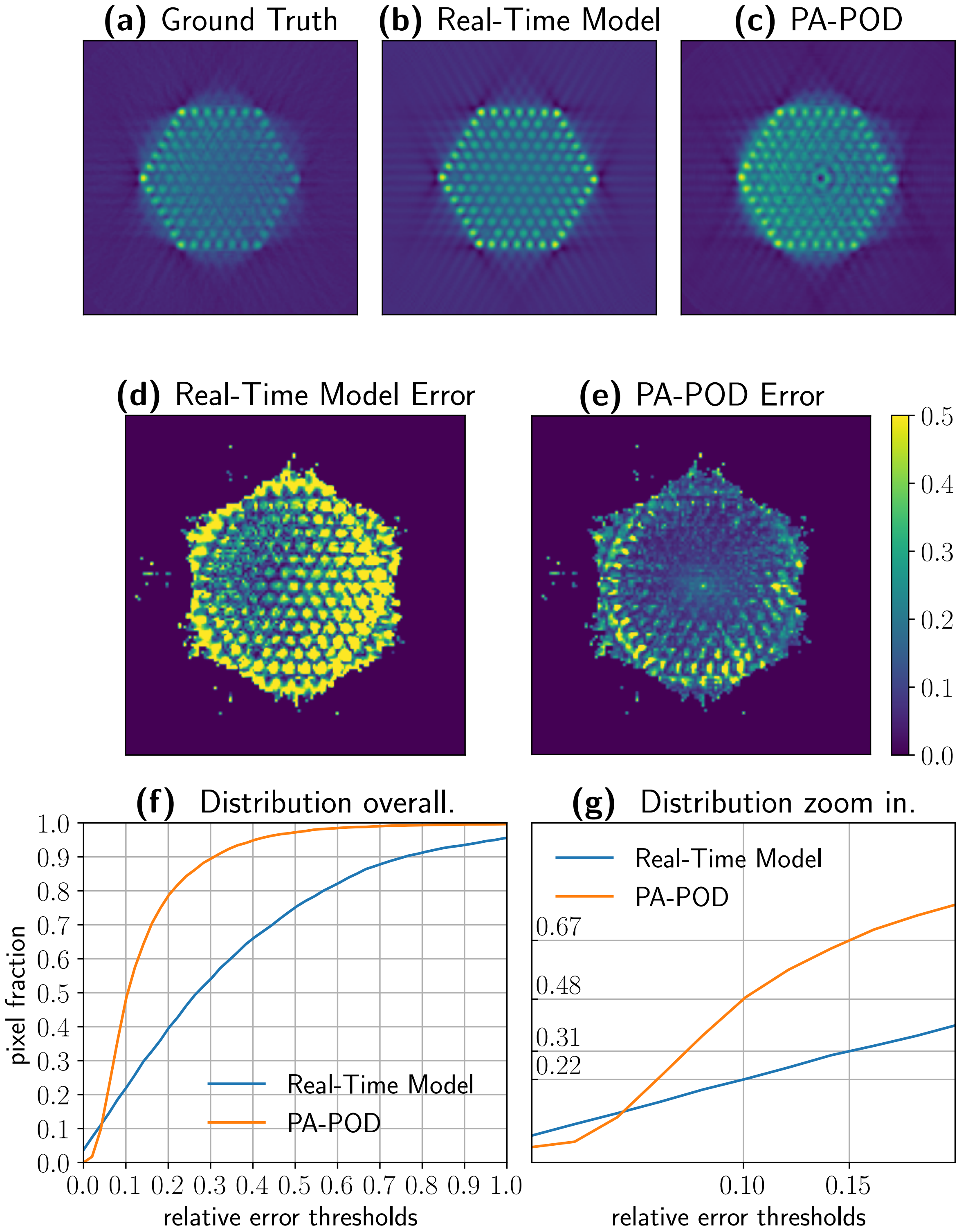

Supplementary Figure 1: **(a)** Ground truth; **(b)** Real-Time Model; **(c)** PA-POD; **(d)** Relative error for the Real-Time Model solution; **(e)** Relative error map for the PA-POD approximated sinogram. In the PA-POD case we randomly sample sixty views of the spent fuel, we repeat the sample one hundred times, and for each pixel, we collect the median of the sampled data. **(f)**: Overall error distribution expressed in pixel fraction; **(g)**: Zoom and highlight on the 10% and 15% error thresholds.

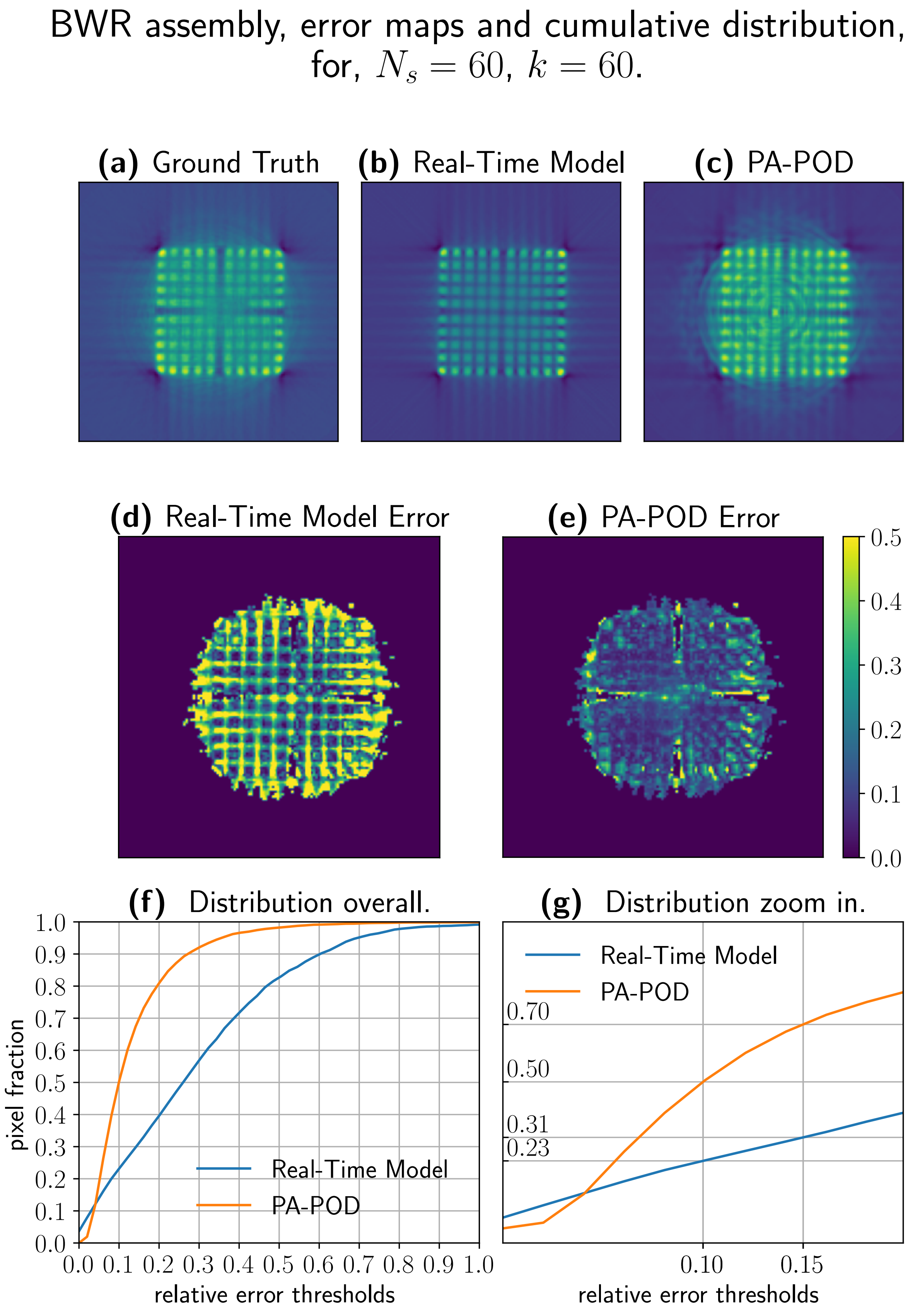

Supplementary Figure 2: **(a)** Ground truth; **(b)** Real-Time Model; **(c)** PA-POD; **(d)** Relative error for the Real-Time Model solution; **(e)** Relative error map for the PA-POD approximated sinogram. In the PA-POD case we randomly sample sixty views of the spent fuel, we repeat the sample one hundred times, and for each pixel, we collect the median of the sampled data. **(d)**: Overall error distribution expressed in pixel fraction; **(e)**: Zoom and highlight on the 10% and 15% error thresholds.
